# Diagnostics of tissue involved injury occurrence of top-level judokas during the competition: suggestion for prevention

**DOI:** 10.7717/peerj.13074

**Published:** 2022-04-04

**Authors:** Wieslaw Blach, Peter Smolders, Jozef Simenko, Krzysztof Mackala

**Affiliations:** 1 Faculty of Physical Education and Sport, University School of Physical Education in Wroclaw, Wroclaw, Polska; 2 European Judo Union, Austria, Vienna, Austria; 3 Essex Pathways Department, University of Essex, Colchester, United Kingdom; 4 Department of Track and Field, Faculty of Physical Education and Sport, University School of Physical Education in Wroclaw, Wroclaw, Polska

**Keywords:** Injury, Tissue, Judo combat, Risk factors, Prevention, Medical survey

## Abstract

**Background:**

Judo, as a high-intensity contact sport, may lead to the occurrence of injuries, especially in competitions. This work aims to assess the likelihood of soft and hard tissue injuries in top-level judokas during competition with defining factors that determine the probability of injury occurrence.

**Methods:**

The injuries that occurred in 123 official international competitions from 2005–2019 were recorded by the European Judo Union (EJU) Medical Commission as a survey that was a part of the EJU Injury Registration form with internal consistency shown by a Crombach Alpha of 0.69. This survey data identified factors such as: sex, anatomical localisation of injury, type of injury, tissue involved and mechanisms of the injury. A total of 650 tissue injuries were reported correctly in terms of tissue injury definition.

**Results:**

The most frequent soft tissue injury (STI) reported was a ligament STI (48.15%), closely followed by skin STI (12.15%) and muscles STI (11.38%). In turn, the most frequent hard tissue injury occurred in bones (8.56%). The highest rates of injuries occurred during the fight in the standing position (78%). Injuries in the standing position mainly occurred while executing a throw (25.85%) and followed by the attempt to throw, *i.e*., the action of reaching the throwing position (22.30%), grip fighting (15.07%), and during falls (14.77%). Opposite to this, fight in groundwork reached only 18.30% soft and hard tissue injuries combined. The ongoing registration of injuries during judo combat and training and the early diagnosis of risk factors for injuries are the basis for the development of effective strategies for injury prevention and further treatment.

## Introduction

Judo, known as the “Way of Gentleness”, is a Japanese martial art and a combat sport developed from *jujutsu* (Kano, 2005). It is comprised of a combination of standing (*e.g.*, throws and takedowns) and ground fighting (*e.g.*, pinning, joint locking, strangulation) without the use of striking techniques (in the competition; however, allowed in kata) (Čierna et al., 2019). It has been reported that 49% of injuries occur to judokas during competitions, 43.60% in training, and 3.7% in physical conditioning (Souza et al., 2006). In competition-oriented judo, the opponent’s resistance plays an important biomechanical role, which may lead to injuries (Frassinelli & Zich, 2014). These injuries in the beginning, without the possibility to know the medical diagnosis, can be classified as a soft injury, medium injury, or serious injury (Pierantozzi & Muroni, 2009).

In recent years, studies in judo concerning injury analysis have been quite frequent (Pierantozzi & Muroni, 2009; Filaire et al., 2001; Frey et al., 2019; Green et al., 2007; Hopkins et al., 2007). However, long-term data rarely appear, and they usually show a different outcome. For example, some research indicated relatively high injury rates in judo, amounting to almost 30% of judokas sustaining injury per competition (Jäggi et al., 2015). However, contrary to these results, an injury study on three consecutive judo competitions showed a much lower percentage of injured judokas with 13.5% (Green et al., 2007) and reported no difference between injury rates among males (41.3 per 1,000 athlete-exposures (A-E)) and females (40.9 per 1,000 A-E). Another research done on high-level athletes during the 2012 London Olympic Games reported an average injury rate of 12.3% in an elite judo competition (Engebretsen et al., 2013), with 12.4% rates for females and 10.9% for male athletes. Extensive research on a large sample of French judokas analysed competition injury incidence, demonstrating an injury incidence of 1.1 injuries per 1,000 A-E per competition (Frey et al., 2019). The overall injury rate for the 2013 World Judo Championships was reported od 47.2 injuries per 1,000 A-E with 38.0 and 52.9 for males and females, respectively (Miarka et al., 2018). Also, a higher prevalence of soft-tissue injuries (58.8%) characterized as stress injuries (36.8%) and miscellaneous traumas (42.6%), mostly occurring during being thrown (29.4%) and gripping fight (32.4%) were reported. A study by Lystad et al. (2021) compared combat sports from three consecutive Olympic games (Beijing 2008, London 2012, and Rio de Janeiro 2016), where judo demonstrated the highest injury incidence rate (IIR) per 1,000 min of exposure with 9.6 injuries per 1,000 min (boxing 9.2, taekwondo 7.7 and wrestling 4.8). However, when the IIR per 100 registered athletes is looked at, judo IIR is the second-lowest with 8.1, just behind wrestling with 5.5, and well under taekwondo 10.9 or boxing 14.5 (Lystad et al., 2021). Research by Čierna et al. (2019) on elite judo athletes looked at the under 23 European Judo Championships in 2015, where they reported the combined IIR per 100 athletes of 8.1 with a higher incidence in females with 9.2 and 7.4 in males. A study on nine Brazilian championships reported that from 2,890 athletes who participated in the tournaments, 16.78% sustained an injury (Machado & Plapler, 2019) and concluded that judo is not a sport with a high incidence of injuries. Research by San Juan (2014) reported injury rates for the men 140.63 per 1,000 A-E and the women 52.24/1,000 A-E, with the upper limbs, being more susceptible to injury in both men and women with injury rates of 70.31/1,000 A-E and 37.31/1,000 A- respectively. A study of 99 elite judo competitions (European Championships, World Championships, Grand Prix, and Grand Slams) during the 2003–2011 period reported a total 3.6% injury rate during the high-level judo events with no differences between genders (Jung, 2017).

It was reported that ACL ruptures and vertebral disc prolapses were the most severe injuries across genders and performance levels regarding time loss and sports performance reduction in judo (Akoto et al., 2018).

Technical-tactical differences between male and female judokas have been shown (Brito, Aedo-Muñoz & Miarka, 2020) in extra light and lightweight women categories as they had shorter accumulated attack times compared with their male counterparts (Sterkowicz-Przybycień, Miarka & Fukuda, 2017). It was reported that in World championships, the percentage of matches completed before the official duration was higher in female categories when compared to males (Ceylan & Balci, 2021). From that, we could assume that female athletes have less exposure time to injuries.

Verhagen & van Mechelen (2009) claimed that sports injuries are diverse in terms of the mechanism of injury, how they present in individuals, and how the injury should be managed. He also claims that trying to precisely define what a sports injury is can be problematic. The definitions are not consistent because they require both theoretical and operational terms discussion. The current study used the definition of an injury proposed by the International Olympic Committee (IOC) manual of sports injuries (Bahr et al., 2012). Therefore, a sports injury may be defined as damage to the body’s tissues resulting from sport or exercise (Bahr et al., 2012; Bahr & Holme, 2003). Therefore in this study, we classified sports injuries about the type of tissues injured, *e.g*., soft tissues (muscle, skin) or hard tissues (bone). There are several signs and symptoms of joint injury, including injury of ligaments and cartilage. A ligament is an elastic band of tissue that connects bone to bone and provides stability to the joint. Injury is recognisable by crackling sound, emerging bruising, and swelling when the injury occurs. Cartilage is soft, gel-like padding between bones that protects joints and facilitates movement. This injury is manifested by a clicking or grinding sensation, stiffness, and lack of swelling-this may not develop for a few hours. When it comes to joint injuries, the main symptoms are a pain when you put weight on the joint, redness, swelling, and lack of instability. Similar symptoms of ligament and cartilage injuries may qualify as joint injury without direct separation. The opinion and classification depend on the doctor examining the injured athlete. In these studies, the classification was made by the EU doctor, who directly looked after the injured judokas.

One of the elements enabling injury limitation during training and competition is estimating components (risk factors) that directly affect the injury. Accurate and individual identification of injury risk factors (Hopkins et al., 2007; Bahr & Holme, 2003; Chen et al., 2005; Franchini et al., 2011) will allow athletes to succeed in judo bouts and competitions. Therefore, injury risk analysis should contain the information on why the athlete is at risk of an injury at a given moment of the fight or training (risk factors) and what injury may arise because of this (Engebretsen et al., 2013; Kim et al., 2015). In general, it was reported that judo athletes sustained 95% of injuries during regular training and only 5% in the competitions (Kim et al., 2015). According to Filaire et al. (2001), it is difficult to determine the possibility of an injury due to the comprehensive, multifactorial impact of numerous factors on the athlete. Therefore, the risk of injuries needs to be assessed. Therefore, knowledge of injury risk factors is essential when we try to avoid injuries and the influence of the direct or indirect critical factors. This is especially important when recognising injury-prone athletes and establishing injury-prevention programs. Therefore, the importance of early recognition of risk factors is even greater.

The study’s main aim is to analyse soft and hard tissue injury incidence among top-level judokas during competitions. The additional objectives were: (a) to indirectly assess the factors that may impact injury appearance; (b) to divide soft and hard tissue injuries by gender; (c) to present injury incidence proportion regarding the body position during combat by gender.

## Materials and Methods

### Study design

This study focuses on identifying risk factors of injuries, analysing injury occurrence among world-class judokas, and determining actions to prevent injuries during competition and training. Information on why a particular athlete may be at risk in a given situation (risk factors) or how injuries may happen was extracted from Wroblewska’s model of potential risk factors (Wroblewska, Stodolka & Mackala, 2020). Afterward, the types and locations of injuries during judo competition were analysed. These data were obtained from the European Judo Union (EJU) Medical Commissioner using a special design questionnaire from 123 competitions carried out between 2005 and 2019. A total number of 650 injuries were analysed. The last step in this study was to identify the potential risk factors and intervention strategies: application of special training and exercises for the prevention of injuries appearing in judo combat. To assess the validity of Wroblewska’s model questionnaire (Wroblewska, Stodolka & Mackala, 2020) and the EJU injury registration form, Cronbach’s alpha was applied. The study followed Brukner & Khan (2012) methods of classifying sports injuries which focuses on understanding the mechanism of injury and the onset of the symptoms.

### Subjects

The data for injury occurrence analysis were collected from a group of 25,397 high-performance class judokas (men and women) that competed in 123 international tournaments (European open tournaments, Grand Prix, Masters, Grand Slam), including senior European Judo Championships. These tournaments were held between 2005 and 2019. The respondents’ sample included one age category: seniors (judokas between 19–35 years old), competing in all judo weight categories. The sample of injury respondents included 650 cases, representing 2.55% of the total number of 14,664 men and 10,733 women competitors registered in all tournaments. The participants were informed of the protocol and procedure of the EJU Injury registration form. Judokas signed informed consent. The EJU Injury registration form was approved by the Medical Commissioner of EJU. The study was approved by the Bioethics Committee at the Regional Medical Chamber (No. 287/KBL/OIL/2020) and accepted by the Human Ethics Committee of the University School of Physical Education in Wroclaw. The minimum sample size for 380 measurements/surveys was needed to achieve a confidence level of 95%, with the real value being within ±9.59%.

### The EJU injury data acquisition

All judokas who sustained an injury during the international competition get medical evaluation and medical treatment if necessary. After the end of the fight, the assessment of the injury and the provided help were recorded by the EUJ doctor with the participation of the competitor in the questionnaire. An injury was defined as the judokas’ physical condition that has temporarily or completely prevented an athlete from continuing competitions at least once in one tournament (between 2005 and 2019). This injury required short medical advice or a visit to the hospital, then treatment and rehabilitation. In the first part of the EJU form, judokas were asked the general information such as sex, grade, and body weight. The second part of the EJU form focused on injury occurrence. Compared to other studies, where competitors filled out injury questionnaires before the competition, our questionnaire was completed immediately after the injury was acquired. This strengthens the credibility of the athlete’s information, which further authenticates the message borne by this report. Thanks to additional records of doctors examining judoka (made on the questionnaire), it was possible to more precisely and thoroughly divide injuries into numerous categories. Based on this information, the medical staff could locate anatomical localisation of injury, type of injury, tissue involved, side of the lesion, and whether the judoka can continue the fight or not. These two elements were used to divide the injury into “minor”, where the necessary help was given to the judokas and he/she could continue the fight. Additionally, after the fight, he/she underwent a short medical and physiotherapeutic treatment so that he/she could continue the tournament. The second category is “serious” injury, where judokas had to get medical help, including transport to the hospital. Such a situation prevents judokas from continuing the tournament and possibly withholds his training for some time if the injury is serious.

The questionnaire (answers of judokas) was divided into two sections/categories concerning the demographic and health factors. Demographics included sex and basic anthropometric information. The health injury factors section contained information about the mechanism of injury, which is directly connected to the technique of movement structure executed during competition. In addition, for better transparency of the collected material, injuries have been assigned to anatomic areas of the body and to the upper and lower body and situation (technical action) in which the injury appeared.

### Statistical analysis

The statistical analysis was performed on the following parameters: gender, type of injury, body regions. Additionally, the soft and hard tissue injury frequency was calculated for the fight action in which the injury occurred. The chi-square test was applied to check if the observed frequencies of the injury incidence in men are similar (match) to the injury incidence in women. Cronbach’s alpha was applied to assess the validity of the EJU Injury Registration form (questionnaire). The significance level was set at a *p*-value < 0.05. Epidemiologic incidence proportion (IP) per 100 athletes was calculated for each gender and the total number of injuries was (Knowles, Marshall & Guskiewicz, 2006). Data analysis was performed using SPSS statistical package version 21.0 (SPSS Inc, Chicago, IL, USA).

## Results

A Cronbach’s alpha of 0.69 was obtained for the EJU registration form. Table 1 characterizes the type of injury which occurred during tournaments. The highest percentage rates reached sprain (43.31%), both: in men and women judokas. The differences between sex were only Δ = 1.22 and were not statistically significant. The contusion comes with 21.38% in second place; however, the differences between men and women showed higher value than the previous injury. Similar differences between genders were also presented in bleeding (Δ = 3.23), indicating more injuries among men. We must always keep in mind that small nosebleeds and superficial skin lesions are not counted since they are not considered an injury. A similar number and percentage of injuries were shown in luxation and unconsciousness for both genders.

**Table 1:** The last step in the forward stepwise selection.

Injury	Number of injuries	%	Men	Women	$\chi^2$	*p*	Incidence proportion (95% CI)		
			No/%	No/%			Men	Women	Total
Sprain	288	43.31	148 (22.76)	140 (21.54)	0.007	0.934	1.01 [0.85–1.17]	1.30 [1.09–1.52]	1.13 [1.00–1.26]
Contusion	139	21.38	81 (12.46)	58 (8.92)	0.015	0.903	0.55 [0.43–0.67]	0.54 [0.40–0.68]	0.55 [0.46–0.64]
Luxation	58	8.92	32 (4.92)	26 (4.00)	0.035	0.852	0.22 [0.14–0.29]	0.24 [0.15–0.34]	0.23 [0.17–0.29]
Unconsciousness	45	6.92	21 (3.23)	24 (3.69)	0.045	0.833	0.14 [0.08–0.20]	0.22 [0.13–0.31]	0.18 [0.13–0.23]
Bleeding	47	7.23	34 (5.23)	13 (2.00)	0.053	0.818	0.23 [0.15–0.31]	0.12 [0.06–0.19]	0.19 [0.13–0.24]
Fracture	27	4.15	16 (2.46)	11 (1.69)	0.077	0.782	0.11 [0.06–0.16]	0.10 [0.04–0.16]	0.11 [0.07–0.15]
Rapture	21	3.23	13 (2.00)	8 (1.23)	0.01	0.751	0.11 [0.03–0.16]	0.11 [0.03–0.16]	0.11 [0.03–0.16]
Commoyio cerebri	19	2.92	11 (1.69)	8 (1.23)	0.011	0.742	0.09 [0.04–0.14]	0.07 [0.02–0.13]	0.08 [0.05–0.12]
Others	6	0.92	4 (0.61)	2 (0.31)	0.038	0.540	0.03 [0.00–0.05]	0.02 [0.00–0.04]	0.02 [0.00–0.04]
TOTAL	650								

Table 2 presents the characteristics of all injuries broken down to soft and hard tissue damage. A total of 650 tissue injuries were filled correctly in terms of tissue injury definition. The most frequent soft tissue injury was ligament (48.15%), closely followed by skin (12.15%) and muscles (11.38). Apart from these values, nerves (8.61%) and joint (6.46%) injuries also reached a high value. In turn, the most frequent hard tissue injury occurred in bones (8.46%). Comparing these five most frequently soft and hard tissue injuries in both genders show that only skin injury showed a statistically significant difference in favor of women (*p* < 0.05).

**Table 2:** Numeric and percentage characteristics of the injury, including gender division.

Type of tissue	Classification		No of injuries	% of all injuries	Men	Women	$\chi^2$	*p*	Incidence proportion (95% CI)		
	soft	hard			No/%	No/%			Men	Women	Total
Ligament	x		313	48.15	158 (24.30)	155 (23.84)	0.006	0.936	1.08 [0.91–1.24]	1.44 [1.22–1.67]	1.23 [1.10–1.37]
Muscles Nerve	x		74	11.38	35 (5.38)	39 (6.0)	0.027	0.869	0.24 [0.16–0.32]	0.36 [0.25–0.48]	0.29 [0.23–0.36]
Ligament Cartilage	x		16	2.0	11 (1.69)	5 (0.77)	0.146	0.703	0.08 [0.03–0.12]	0.05 [0.01–0.09]	0.06 [0.03–0.09]
Brain	x		11	1.69	7 (1.07)	4 (0.61)	0.196	0.658	0.05 [0.01–0.08]	0.04 [0.001–0.07]	0.04 [0.02–0.07]
Skin	x		76	12.15	26 (4.0)	50 (7.69)	0.029	0.864	0.18 [0.11–0.25]	0.47 [0.34–0.59]	0.30 [0.23–0.37]
Bone		x	55	8.46	29 (4.46)	26 (4.0)	0.037	0.849	0.20 [0.13–0.27]	0.24 [0.15–0.34]	0.22 [0.16–0.27]
Joint	x		42	6.46	22 (3.38)	20 (3.07)	0.048	0.827	0.15 [0.09–0.21]	0.19 [0.11–0.27]	0.17 [0.12–0.22]
Nerve Cervical vertebrae	x	x	56	8.61	27 (4.15)	29 (4.61)	0.036	0.850	0.18 [0.11–0.25]	0.27 [0.17–0.37]	0.22 [0.16–0.28]
Nail Cornea Tympanum	x x	x	4 2 0	NA NA NA	1 0 0	3 0 0	NA NA NA				
Others			4	NA	1	4					
Total			650								

Table 3 presents that the highest injury percentage rates concerned the fight in the standing position (78%), then 18.30% in ground combat. The illegal move has the lowest percentage of injuries, only 3.69%. In turn, in the fight in a standing position, the injuries were mainly in the action of performing the throw (25.85%). There were no statistical differences between men and women. The judokas (both men and women) experienced muscle tears, sprain of the joints, ligaments damage, or broken fingers and bone. In turn, fight in groundwork reached only 18.30% of soft and hard tissue injuries. The highest rates appeared in the armlock position (8%) and in the strangle position (6.12%).

**Table 3:** Numeric and percentage characteristics of the soft and hard tissue injury, including gender division.

Body position during combat	Type of injury – soft tissue	Type of injury-hard tissue	Participant	No of injuries	% of all injuries	Men	Women	$\chi^2$	*p*	Incidence proportion (95% CI)		
						No/%	No/%			Men	Women	Total
***Standing position***												
Grip Fighting	abrasion of the epidermis, muscle tear dislocation of the joints of the phalanges	broken fingers	both judokas	98	15.07	57 (8.77)	41 (6.31)	0.021	0.885	0.39 [0.29–0.49]	0.38 [0.27–0.50]	0.39 [0.31–0.46]
Attempting throw	muscle tearligament damage sprain of the joints	N/A	attacking judoka	145	22.30	60 (9.23)	85 (13.08)	0.014	0.905	0.41 [0.31–0.51]	0.79 [0.62–0.96]	0.57 [0.48–0.66]
Being throw	joint sprains muscle tear	N/A	performing throw judoka	168	25.85	83 (12.77)	85 (13.08)	0.012	0.913	0.57 [0.44–0.69]	0.79 [0.62–0.96]	0.66 [0.56–0.76]
Fall Counter-attack	contusions concussion damage to the cervical spine	bone fractures cartilage damage	throw judoka	96	14.77	49 (7.54)	47 (7.23)	0.021	0.885	0.33 [0.24–0.43]	0.44 [0.33–0.54]	0.38 [0.28–0.48]
***Groundwork***												
Hold-down	abrasions of the epidermis muscle tears joint sprains	N/A	hold judokas	27	4.15	13 (2.00)	14 (2.15)	0.074	0.785	0.09 [0.04–0.14]	0.13 [0.06–0.20]	0.11 [0.07–0.15]
Strangle	loss of consciousness cervical spine injury hematoma	fracture	hold judokas	40	6.15	17 (2.62)	23 (3.54)	0.051	0.821	0.12 [0.06–0.17]	0.21 [0.13–0.30]	0.16 [0.11–0.21]
Armlock	fructure muscle tear sprain of the elbow joint		both judokas	52	8.00	27 (4.15)	25 (8.85)	0.039	0.844	0.18 [0.11–0.25]	0.23 [0.14–0.32]	0.20 [0.15–0.26]
***Other***Illegal move	fingers in the eye blow to the nose twist the wrist finger grip	N/A	both judokas	24	3.69	10 (1.54)	14 (2.15)	0.086	0.770	0.07 [0.03–0.11]	0.13 [0.06–0.20]	0.09 [0.06–0.13]
Total				650	650		316	334		2.15 [1.92–2.39]	2.28 [2.00–2.56]	1.42 [1.27–1.56]

## Discussion

### Diagnostics of tissue involved injury occurrence

The present study analysed soft and hard tissue injury incidence among Europe’s top-level judokas during competitions. An additional goal was to indirectly assess the factors that may have an impact on injury appearance. Cronbach Alpha for the EJU questionnaire was reported in the acceptable range of 0.69.

Recently, some studies have analyzed the injuries in the elite men and women judokas (Green et al., 2007; Engebretsen et al., 2013; Kim et al., 2015; Maciejewski & Callanta, 2016) but little information is posted on soft and hard tissue damage injuries. The entire mechanism of injury in judo is associated with the technical execution of individual combat elements, divided into a standing position (gripping, attempting a throw, being thrown, or fall after being thrown) and groundwork (hold downs, leverages and choking or getting out or defend from/of these situations).

The studies of Ruddy et al. (2019) and Bittencourt et al. (2016) indicated that injuries occur due to complex and non-linear interactions between multiple factors. In turn, Bahr & Holme (2003) claimed that even one isolated factor can strongly impact injury occurrence or is capable of sowing enough information to predict these injuries but only at the individual level. This information directs us to suppose that injuries may be caused by elements related to the technique-the performance of a given movement structure, performed in a different phase (situation) (Hopkins et al., 2007) and spatial position of the body during judo combat. Therefore, it is essential to determine the starting position in which the action occurs when performing a specific throw. According to our analysis, most injuries concerned the fight in the standing position (78%). Results from our study are similar to the research conducted by Green et al. (2007) and Pierantozzi & Muroni (2009), who also showed that the majority of injuries in the standing fight position affect the upper limbs. It seems logical because the judoka needs to grip the opponent before the throwing movement begins. However, this was not confirmed in our research, which indicates that attempt to throw, *i.e*., the action of reaching the throwing position (22.30%) reached a bigger value than gripping. Furthermore, in this body position of judo combat, female judokas showed a statistically significant (*p* < 0.05) greater number of injuries than men (Table 4). In turn, the gripping action was more injurious for men. The gripping and attempting throw of the opponent required constant movement of the upper and especially lower extremities. This is probably because more time is spent in standing combat, where judokas must grip their opponent before executing the attack (Pierantozzi & Muroni, 2009; Hopkins et al., 2007). This indicates the performance of very dynamic movement structures, with the lower limbs’ dynamic work, which constantly changes the position and direction of the move. This increases the risk of soft tissue injury, especially ligament damage (48.15% of all injuries), followed by muscle injuries (11. 38%) (Table 4). It must be remembered that the action of trying to throw is linked with the opponent’s action to throw the first judoka. In this action, we noticed 25.85% of soft tissue injuries that mainly occurred as joint sprains and muscle tears.

**Table 4:** Wroblewska’s model of the risk of injury.

Step 9	LOGIT	Model creating Distribution: BINOMAIL, LINK Function: LOGIT Modeled probability of injure = 1					Model creating
Effect		df	Wald Stat.	Wald p	S. pkt Stat.	S. pkt p	
Previous injuries Age Sleep Blood Competition Training Load At. conditions		3 4 1 1 3 4 3	40. 6608 18.3029 10.2730 2.5473 4.709659 9.674838 9.082616	0.000000 0.001077 0.001350 0.110478 0.194334 0.046276 0.028212	– – – – – – –	– – – – – – –	In model In model In model In model In model In model In model

The action being thrown ends with landing on the mat, where we also recorded a high injury rate in both soft and hard tissue injuries (14.77% of all injuries). Our study has certain similarities with other studies; however, they vary in some details (Pieter & De Crée, 1997; Pieter et al., 2001; James & Pieter, 2003; Murayama et al., 2014). The emerging inaccuracies most likely result from the fact that our analysis took into account soft and hard tissue injuries and not, for example, the type of injury in general or injuries to the body’s parts. Judokas appear to sustain these injuries at similar rates regardless of gender. Additionally, our analysis showed that female judokas reach higher rates of injuries in groundwork action, and this is the case of hold-downs and strangle positions (Hopkins et al., 2007; Pieter et al., 2001). The main soft tissue injuries include: previously mentioned muscle tears, joint sprains, and additional loss of consciousness (6.29%), cervical spine injury (2.29%), and abrasions of the epidermis. In turn, the hard tissue injury only appears as the fracture (Table 3). Concussions (commotion cerebri) were diagnosed only 19 times, and in four instances, the judoka had to be transported to a hospital. A concussion is defined as an injury resulting from hitting the mat during a fall or being choked by an opponent (Green et al., 2007; Akoto et al., 2018; Murayama et al., 2014). It prevents the judoka from interrupting the fight by himself (to tap out); for the safety and life of the judoka, the referee must immediately react and stop the fight (Nambu & Noji, 2014) to prevent any serious consequences.

The major hard tissue injury among judokas in our study was a fracture, with 27 cases; however, in 23 cases, it was considered a very serious injury that included hospitalization and further rehabilitation. The most frequently fractured area reported was the hand fracture, followed by a clavicle fracture and the cervical fractures (Green et al., 2007). A cervical spine fracture, commonly called a broken neck, can occur secondary to exaggerated flexion or extension or because of direct trauma or axial loading. In turn, clavicle fractures are most often caused by a direct fall onto the shoulder.

### The probability of injury occurrence

An important factor in understanding injuries in judo and other sports is knowing the risk factor of their occurrence (Hopkins et al., 2007; Bahr & Holme, 2003; Knapik et al., 2001). It may be a direct or indirect influence. This mainly applies to the general problem of injury in sport, but with a special focus on how to reduce it (Wroblewska, Stodolka & Mackala, 2020; Ruddy et al., 2019; Bittencourt et al., 2016). It was shown that injuries result from complex and non-linear interactions between multiple factors (Ruddy et al., 2019; Bittencourt et al., 2016). Going forward, Bahr & Holme (2003), in their research, indicated that even one isolated factor can strongly impact injury occurrence or is capable of sowing enough information to predict these injuries but only at the individual level. From a group of twenty-four factors (Wroblewska, Stodolka & Mackala, 2020), the seven most important factors: previous injuries, age, sleep, blood, competition, training load, and atmospheric conditions impacting the risk of injury were selected for future discussion. The last factor goes beyond the spectrum of a single sport. These injuries risk actors can be attributed to almost every sport as they are so highly utilitarian.

Judo presents one of these sports where the above-mentioned risk factors could be applicable to identify potential risk factors for the occurrence of injuries in judo. Wroblewska model (21) risk factors have been shown to individually affect judo performance (previous injuries (Sacras, Ribeiro & M, 2020), age (Frey et al., 2019; de Carvalho et al., 2018), sleep (Souissi et al., 2013; Dunican et al., 2017), blood (Degoutte, Jouanel & Filaire, 2004; Murata et al., 2016; Casals et al., 2017; Türkmen, 2011; Obmiński, Lerczak & Błach, 2010), competition (Frey et al., 2019; de Carvalho et al., 2018), training load (de Carvalho et al., 2018; Manzato et al., 2017) and atmospheric conditions (Obmiński, Lerczak & Błach, 2010; Tomazin et al., 2021; Almeida et al., 2021)), however, until now, they haven’t been grouped in one study. Therefore, the usage of this method presents a novelty in judo in an exploration of potential risk factors that could directly or indirectly influence the occurrence of injuries in judo. The first step toward injury evaluation *via* the modeling process was by selecting the factors that influence expected injuries and establishing relations between them. Although these factors do not originate directly from the EJU questionnaire, subjecting it to direct analysis in conjunction with injuries occurring during judo combat will bring a different look at the completely different injury assessment face. Furthermore, this model should be additionally explored and possibly updated, as research shows that hydration and weight cycling are considered important injury risk factors in combat sports (Pocecco et al., 2013).

Most judokas reduce their weight quickly before the competition by introducing fluid restriction, sauna or plastic clothing, diuretics, or food restriction (Artioli et al., 2010; Brito et al., 2012; Langan-Evans, Close & Morton, 2011; Lakicevic et al., 2020). Rapid bodyweight reduction (dehydration in 12 to 96 h), typically with fluid restriction and increased exercise, is used by athletes competing in weight-class events. During bodyweight loss, slow glycogen resynthesis after training, loss of muscle protein, and stress fractures (caused by endocrinological disorders) may affect competition and increase the risk of injury (Fogelholm, 1994). To summarise this paragraph, we need to follow the Filaire et al. (2001) statement, which claims that judokas need to be strongly advised not to reduce the weight by more than 5% before a competition. More significant body mass reduction puts judokas at a greater risk of injury.

Sleep deprivation reduces the ability to react quickly and think clearly, which causes athletes, including judokas, to be exposed to making poor decisions and taking high risks during combat. A lack of sleep also increases irritability and the risk for anxiety and depression. This can result in injury exposure during a fight in different combat actions (Fullagar et al., 2015; Bonnar et al., 2018; Pedlar, Newell & Lewis, 2019).

Another risk factor of injury can be found in blood markers. Blood tests are now becoming more important and necessary action in high-performance sports settings as a physiological profiling and monitoring tool. A range of biomarkers can provide information relating to athlete readiness to train, including biomarkers of oxidative stress (OS), inflammation, protein turnover, and hormones (Pedlar, Newell & Lewis, 2019; Crewther et al., 2011). It is also essential to have data from blood, which determine whether competitive exercise aggravates markers of muscle fiber damage and delays the recovery of performance and muscle glycogen stores (Baird et al., 2012; Nybo et al., 2013). Thanks to the information (bio-markers) from the blood, we may indicate the efficacy of training intervention, the tolerance of training load, or nutritional strategies.

The more the athlete trains and competes, the more he/she is exposed, and with this, more different types of injuries may occur. The training methods these days emphasize more on motor ability development than personal skill–technique. This approach may be responsible for that high level of injury and associated anatomical locations among judokas. Better motor preparation of the judo participants (Amtmann & Cotton, 2005; Drapšin et al., 2009; Pocecco et al., 2012; Frassinelli, Niccolai & Zich, 2017; Mackala et al., 2019) enhances the application of more powerful structure-new maneuvers, which may broaden and increase the types of injuries. This increases their power and speed, resulting in stronger impacts during full-body contact.

Furthermore, increased exposure to full-body contact elevates the risk of injuries. Chen et al. (2005) summarised that the frequency and number of injuries, as well as the significance of the injury, further influences the training and the resulting competitions. These all training elements are linked to the age factor. It applies to both the younger/less experienced competitor and the older/more experienced judoka. This means that an inexperienced fighter is more prone to injuries during a fight due to the still weaker motor preparation and technical skills, *e.g*., the ability to fall after a throw.

On the other hand, an older judoka/ more experienced, despite better technical skills, is also at risk of injuries. They may result from overtraining, and above all, recklessness and lack of concentration in the fight against a weaker fighter. Surprise-a sudden grab and throw of the opponent causes a lack of preparation for landing and an increased risk of injury during a fall. However, according to Green et al. (2007), judokas with less experience were injured at a rate of 14,6% when compared to more experienced judokas with 21.9%. In turn, Frey et al. (2019) claimed that lower-level competitions compared to higher ones showed a higher frequency of injuries. Moreover, judo combat with a high difference in the competitors’ performance level pointed to a higher frequency of injuries.

### The suggestion for prevention of injury occurrence

Nobody can 100% predict the occurrence of an injury during a judo fight. It is caused by too many factors that may directly or indirectly be related to the occurring injury. In judo, where there are constant changes of actions with applications of different movement structures, the ability to be at the right time, in the right position, to optimize attack or defense action strategies, and improving the effectiveness of the adopted tactical scheme is of primary importance to avoid injuries. According to the classification of van Mechelen, Hlobil & Kemper (1992), the injury risk factors are mostly divided into two main categories: internal-athlete-related risk factors, often recognized as intrinsic factors, and external-environmental risk factors. Both categories are relevant, but in the case of suggestions for prevention, the categories internal-athlete-related risk factors take on greater importance. Therefore, knowledge of the emergence of judo injuries is essential to develop preventive measures. The main focus must be on profiling and monitoring. Measuring blood biomarkers can help athletes avoid injury and disease by adjusting their diet, training load, and recovery strategies. Judokas and their coaches must avoid harmful weight loss procedures before the competition (Langan-Evans, Close & Morton, 2011; Fogelholm, 1994). This is to be prevented by special programs informing about the harmfulness of this precedent. Proper weight control through the use of an appropriate diet can largely eliminate rapid weight loss and reduce the related injuries during the period preceding the competition. An important additional issue in judo training and, first of all, in a competition, is applying a well-balanced approach to training technique and motor preparation, combined with combat strategy (Mackala et al., 2019). This allows to efficiently use strength and power capacity and coordination skills to increase motor performance effectiveness (Frassinelli & Zich, 2014).

Also, appropriate testing of the training effect, including technical skills, especially in less experienced judokas, might help evaluate and reduce the risk of injury, mainly during falls (Kalina et al., 2008). As beforementioned, new testing solutions should be introduced into judo training to identify risk factors and help implement preventive measures. One of those tests could be the Functional movement screen (FMS), as it fits perfectly in this description as it checks functional movement asymmetries between different parts of the body and, from the total score, gives an assessment of movement, which helps coaches to prevent the occurrence of injuries and includes a system of preventive exercises (Simenko, 2019; Ciz et al., 2017). Also, the regular usage of testing batteries, which include measurements of the most important joint muscles around frequently injured joints, like isokinetic (Kons, Detanico & Franchini, 2020; Šimenko, Karpljuk & Hadžić, 2022) tensiomyography (TMG) (Monteiro et al., 2016; Garcia et al., 2011), body composition through electrical bioimpedance (weigh and body composition management), or even modern 3D body scanners (Simenko et al., 2017) is advised for prevention strategies of elite judokas. Recently it was reported that the isokinetically measured DCR (dynamic control ratio) ratio with the eccentric Hamstrings strength could also help in the early identification of vulnerable judokas and help in optimal conditioning of judokas (Šimenko, Karpljuk & Hadžić, 2022). Also, raising awareness with the regular implementation of already available exercise prevention programs developed especially for judo (Akoto et al., 2018; Malliaropoulos et al., 2019; von Gerhardt et al., 2020) would be helpful.

Based on the above assumptions, we can conclude that our hypothesis that fewer hard tissue injuries will occur during female judo combat was not confirmed. The statistically significant differences (*p* < 0.05) between male and female judokas occurred in the gripping (in favor of men) and in the attempt of throwing phase (in favor of women). Injuries in the remaining stages of the fight showed almost equal values. In turn, the analysis of the types of injuries, with detailed data on soft and hard tissue injury, showed that the most frequent soft tissue injury was ligament (48.15%), closely followed by skin (12.15%) and muscles (11.38%). In contrast to this, the most frequent hard tissue injury occurred in bone (8,56%). The highest percentage rates concerned the fight in the standing position (78%), which was dominated by the action of performing the throw only (25.85%) and attempt to throw, *i.e*., the action of reaching the throwing position (22.30%). The grip fighting was third, with an incidence of 15.07%, and the fourth was the fall (14.77%).

### Limitations

Although most injury risk factors analyzed in this study do not originate directly from the EJU questionnaire but subjecting it from athletes to direct analysis in conjunction with injuries occurring during judo combat will bring a different look at the completely different face of injury assessment, followed by the use of proper medical treatment and rehabilitation. Also, the rules in judo competitions changed from 2005 to 2019, and the effect of different time-motion structures was not considered in the injury analysis. Furthermore, the injury analysis was not carried out by specific weight categories for males and females, which could give us additional insight into injury occurrence and possible prevention. Additionally, the athlete exposure time as the number of fights per competition and minutes was not gathered, which disabled us to calculate the Incidence rate or injury incidence per 1,000 athlete exposures. Based on that, we recommend that the medical commission update the injury registration form to include the aforementioned data. Finally, the data was collected only from the competition. Therefore, future studies should analyse the injury incidence proportion regarding the body position during combat by gender weight categories. Additionally, the same should be investigated in junior and cadet age groups to identify the most common injuries, mechanisms, and risk factors, which could help in lowering the youth athletes’ dropout rates.

## Conclusion

The present study shows comprehensive knowledge of the frequency and characteristics of soft and hard tissue injuries in judo. Additionally, we specifically indicate the judoka’s body’s temporal and spatial position during combat, where injuries occurred, with the highest injury occurrence in motions; being thrown (26%), attempting to throw (26%), and grip fighting (15%). The highest proportion by injury tissue type was the ligament injuries (48%), followed by skin (12%) and muscles (11%). The most common injury was the sprain (43%), followed by contusions (21%) and luxation (9%). The study also provides an additional point of view about injury risk factors during judo activity. These factors represent an essential basis for developing effective strategies for injury prevention and further treatment. Therefore, it is of great importance to introduce systemic and constant monitoring of injuries in judo and the factors causing them. Our analysis indicates that early identification of risk factors and their gradation will help prevent further injuries in training and combat.

The ongoing registration of injuries during judo combat and training and the early diagnosis of risk factors for injuries are the basis for developing effective strategies for injury prevention and further treatment.

## Supplemental Information

10.7717/peerj.13074/supp-1 Supplemental Information 1 Raw data.

## Additional Information and Declarations

### Data Availability

The following information was supplied regarding data availability: The raw measurements are available in the Supplemental File.
